# Supplementary material for: Functional differences in seasonally absorbed nitrogen in a winter-green perennial herb
Source: R Soc Open Sci. 2020 Jan 29;7(1):190034. doi: 10.1098/rsos.190034 (PMC7029918; doi:10.1098/rsos.190034)
Supplement: Supplementary figure and table [file rsos190034supp1.doc]

*Nishitani et al. “Functional differences in seasonally absorbed nitrogen in a winter-green perennial herb” Royal Society Open Science*

**Fig. S1** Soil temperature (from June to September: the leafless period of *Lycoris radiata* var. *radiata*) and air temperature (from October to April) during the period of (A) cultivation in an experimental garden at Tokyo Metropolitan University (TMU) and (B) fertilizer supply experiments at Nippon Medical School (NMS). The soil temperature was measured in the middle of a pot (at a depth of approximately 5 cm), and the air temperature was measured at the highest point of the plants’ leaves (approximately 3 cm above the ground).

**Table S１**

Leaf nitrogen (N) concentration in *Lycoris radiata* var. *radiata* plants treated with fertilizer at different times (see Fig. 1).

Leaf materials were collected on 25 Mar. 2011. The values are means, with 1 s.e. in parentheses (*n* = 6). Different lowercase letters represent significant differences among the fertilizer supply treatments (*P* < 0.05, Games-Howell’s method for multiple comparisons).
