# Supplementary material for: Functional differences in seasonally absorbed nitrogen in a winter-green perennial herb
Source: R Soc Open Sci. 2020 Jan 29;7(1):190034. doi: 10.1098/rsos.190034 (PMC7029918; doi:10.1098/rsos.190034)
Supplement: Data for biomass, concentrations of nitrogen and pigments, and leaf phenology [file rsos190034supp2.pdf]

Data for biomass, concentrations of nitrogen and pigments, and leaf phenology

FW: total plant fresh weight (g) in April 2010
LA: leaf area (cm^2) at harvest
DW: dry weight (mg) of each organ at harvest
N, P, O: new scale, previous years scale and scale older than previous years', respectively, of the bulb
%N: nitrogen concentration (%) of each organ at harvest

PC: pigment concentration （μmol m^{-2}）
Neo:neoxanthin　Zea: zeaxanthin
Vio: violaxanthhin　Chb: Chlorophyll b
Ant: antheraxanthin　Cha: Chlorophyll a
Lut: lutein　β: β carotene

No	Treatment	FW	LA	DW leaf	DW N	DW P	DW O	DW Root	%N leaf	%N N	%N P	%N O	%N Root	PC Neo	PC Vio	PC Ant	PC Lut	PC Zea	PC Chb	PC Cha	PC β
1	C0	5.82				391.2	1071.4	67.9			0.78	0.55	1.42								
2	C0	5.66				375.5	1310.3	36.1													
3	C0	5.33				280.1	1188.7	52.1			0.65	0.41	1.27								
4	C0	5.44				266.9	1214.9	96.3													
5	C0	6.60				401.3	1386.5	65.1													
6	C0	6.78				386.0	1477.2	111.7			0.7	0.41	1.24								
7	C0	5.96				393.7	1294.5	65.6													
8	C0	5.15				242.4	1265.8	50.4													
9	C0	5.40				295.0	1147.8	92.2													
10	C0	5.53				390.8	1207.2	76.0													
11	C0	6.18				370.4	1348.8	89.1			0.58	0.34	1.25								
12	C0	6.10				343.1	1350.7	76.2													
13	C0	6.31				333.1	1352.9	109.0													
14	C0	5.09				246.2	1171.5	53.7													
15	C0	6.01				380.2	1308.2	39.4													
16	C0	5.75				348.8	1297.7	48.5			0.71	0.45	1.55								
17	C0	6.44				306.8	1362.1	86.9													
18	C0	6.65				387.8	1463.2	68.5													
19	C0	5.86				290.7	1106.8	125.1													
20	C0	6.83				376.2	1591.6	42.9			0.65	0.51	1.59								
101	C1	5.02	10.0	81.5	124.7	423.8	894.6	120.6	2.76	0.530	0.204	0.195	1.10	22.70	14.04	18.16	82.88	37.57	153.5	342.3	52.14
102	C1	5.12	16.1	132.3	176.2	472.0	801.8	184.0	2.80	0.537	0.191	0.225	1.29	26.33	12.15	17.45	84.18	23.08	196.1	405.7	47.39
103	C1	5.28	17.4	140.9	181.1	596.5	1001.8	117.1													
104	C1	5.41	10.1	81.8	151.8	439.1	690.3	193.4						28.47	18.48	23.39	99.26	40.17	177.5	431.8	60.11
105	C1	5.53	10.7	87.4	133.7	483.0	1042.0	140.2						23.63	15.79	21.64	90.93	39.17	148.8	341.8	51.69
106	C1	5.54	6.6	49.9	103.3	393.9	820.4	160.6													
107	C1	5.72	13.3	109.9	192.9	590.8	729.2	180.8						25.44	12.86	16.89	88.38	35.91	169.6	411.6	55.51
108	C1	5.78	27.6	223.3	243.3	604.2	1134.8	211.0													
109	C1	5.78	11.2	88.7	192.9	546.1	789.9	168.2													
110	C1	5.90	8.2	66.0	125.4	548.0	877.6	142.2						22.56	10.94	15.74	88.16	35.94	162.7	349.6	50.63
111	C1	5.92	9.8	79.9	107.9	436.6	1015.2	159.7	2.53	0.494	0.214	0.180	1.20	23.71	13.30	16.73	90.53	39.71	161.6	356.9	49.64
112	C1	6.02	21.7	175.2	272.6	621.1	520.2	297.5													
113	C1	6.12	21.5	174.0	273.1	850.8	992.2	121.9													
114	C1	6.18	9.6	78.0	189.5	756.5	633.2	204.0	2.78	0.427	0.229	0.235	1.01	25.52	15.57	19.13	96.26	34.87	170.6	375.1	54.52
115	C1	6.32	29.0	234.3	284.4	988.5	916.6	209.1													
116	C1	6.41	14.0	114.1	240.4	739.5	848.6	187.5						23.66	12.43	18.69	84.91	37.28	160.5	349.8	55.42
117	C1	6.58	15.1	121.8	185.9	616.5	1053.5	182.6													
118	C1	6.65	17.0	138.1	196.6	550.1	973.0	184.4						25.02	11.81	14.31	86.49	42.39	173.1	370.7	55.69
119	C1	6.77	18.0	145.6	238.3	745.9	858.6	210.3													
120	C1	6.80	19.8	157.0	185.9	527.8	965.3	277.0	2.87	0.539	0.197	0.225	1.37	27.59	11.16	15.78	97.10	46.90	190.1	403.7	59.83
121	C1	5.42	9.3	75.9	126.7	402.0	720.2	152.7	2.85	0.574	0.194	0.195	1.36	24.60	11.18	16.25	83.71	37.91	169.3	407.5	57.00

Treatment No	FW	LA	DW leaf	DW N	DW P	DW O	DW Root	%N leaf	%N N	%N P	%N O	%N Root	PC Neo	PC Vio	PC Ant	PC Lut	PC Zea	PC Chb	PC Cha	PC β
201 Au	5.07	28.6	251.0	257.3	574.7	783.0	188.3													
202 Au	5.15	30.5	267.7	420.8	733.3	639.6	195.2													
203 Au	5.21	13.2	120.0	208.9	653.9	668.8	138.9	3.20	0.580	0.318	0.329	1.48	30.65	21.43	24.22	113.9	36.87	209.5	460.0	79.23
204 Au	5.37	29.7	248.3	334.3	749.6	702.1	155.5													
205 Au	5.49	15.0	133.5	241.4	504.4	787.1	152.1	3.30	0.752	0.337	0.369	1.65	35.00	19.87	22.05	126.7	47.42	255.1	548.8	93.35
206 Au	5.59	10.1	89.5	158.1	523.0	741.1	168.8						35.45	23.72	21.63	123.9	38.53	243.6	588.8	85.63
207 Au	5.70			197.7	401.2	882.5	182.8						38.70	24.79	24.59	137.9	33.81	288.3	589.3	96.27
208 Au	5.74	21.3	181.5	235.3	535.3	946.3	213.6						35.17	15.34	20.00	126.0	57.57	253.8	536.2	84.97
209 Au	5.82	27.1	237.4	255.1	862.3	736.9	176.9													
210 Au	5.85	16.4	149.5	238.2	436.9	548.9	251.0													
211 Au	5.99	29.6	253.2	363.1	612.8	792.3	218.2	3.50	0.615	0.363	0.388	1.79	34.31	14.98	23.78	119.4	52.61	238.8	498.1	72.19
212 Au	6.06	15.1	133.8	296.3	613.8	790.4	190.9	3.08	0.533	0.310	0.269	1.25	34.47	29.58	23.58	124.5	28.85	242.7	594.2	88.05
213 Au	6.07	24.3	214.4	352.8	738.4	590.5	268.2						31.16	16.46	21.42	114.4	53.11	227.5	483.9	72.36
214 Au	6.24	13.2	120.3	237.5	541.3	862.5	187.2						34.56	29.10	32.12	122.0	45.42	229.2	555.7	82.33
215 Au	6.30	25.3	221.7	307.1	641.9	839.9	240.0						35.12	16.82	23.65	122.7	52.87	249.0	519.2	76.78
216 Au	6.53	21.0	184.1	245.7	657.8	1248.8	169.0													
217 Au	6.54	22.5	194.5	319.8	894.9	800.7	276.1	3.38	0.486	0.293	0.320	1.67	31.91	20.56	21.45	112.5	34.00	225.4	480.4	87.80
218 Au	6.67	18.2	151.7	261.2	629.8	948.9	203.8	3.21	0.559	0.304	0.286	1.47	26.40	15.67	19.68	87.69	28.41	198.7	417.4	58.61
219 Au	6.78	23.2	203.4	307.6	760.9	1037.0	280.2													
220 Au	6.95	31.9	279.5	387.2	847.1	1091.8	368.4													
221 Au	6.04	19.0	166.7	204.7	524.0	1163.8	155.5													
301 Su	5.01	34.1	283.4	410.7	1113.5	584.9	195.6													
302 Su	5.13	25.5	223.1	385.7	590.2	768.9	221.1	3.26	0.468	0.303	0.319	1.45	27.70	13.54	16.09	102.2	41.84	200.4	437.5	67.71
303 Su	5.22	32.6	281.5	521.0	688.0	647.3	247.4	3.28	0.478	0.318	0.386	1.57	32.35	18.24	19.31	117.8	50.62	234.9	494.2	76.87
304 Su	5.37	29.9	249.0	411.1	777.3	926.2	164.7													
305 Su	5.43	18.2	156.6	349.6	748.8	532.1	195.5						27.76	19.06	21.35	100.4	37.82	192.0	413.4	61.78
306 Su	5.62	41.0	341.0	629.4	798.0	709.9	286.3													
307 Su	5.71	38.4	319.6	587.9	856.8	607.8	287.5													
308 Su	5.75	23.9	205.5	445.2	702.9	660.3	222.5						30.03	18.23	22.80	105.4	35.75	197.8	426.6	62.42
309 Su	5.79	35.2	309.9	479.1	711.7	860.5	273.1						32.57	13.40	18.44	113.0	55.38	217.7	525.5	79.49
310 Su	5.83	29.2	239.8	401.8	781.5	1030.0	229.6	2.85	0.367	0.218	0.225	1.30	29.02	10.86	16.49	96.9	38.94	205.7	432.4	59.67
311 Su	5.92	33.5	281.2	581.4	871.0	664.3	234.0	3.13	0.415	0.304	0.362	1.43	29.19	19.04	21.67	107.7	40.78	204.8	435.6	62.18
312 Su	6.06	32.8	280.3	575.1	764.7	758.5	184.5						33.26	24.29	25.14	116.3	32.00	224.4	540.9	77.40
313 Su	6.17	29.0	236.1	448.8	781.4	748.6	206.4						28.85	13.96	19.32	105.3	49.32	196.2	427.4	60.31
314 Su	6.19	36.8	306.3	401.8	760.6	1190.4	244.3													
315 Su	6.29	36.2	295.1	498.2	773.4	1020.3	270.3						29.93	11.46	17.55	110.5	56.87	205.3	435.8	65.26
316 Su	6.53	26.3	218.9	418.7	923.2	977.1	191.6													
317 Su	6.57	24.1	194.8	347.8	715.6	833.7	208.3	2.96	0.443	0.251	0.247	1.33	29.95	28.68	24.89	107.8	32.19	189.2	407.6	63.61
318 Su	6.67	19.1	136.3	226.7	660.3	1036.5	206.3													
319 Su	6.71	39.1	325.5	588.9	1122.9	884.2	299.0													
320 Su	6.86	30.8	248.6	507.0	817.2	954.5	195.5	3.05	0.367	0.218	0.257	1.37	26.27	22.00	18.95	100.8	35.55	182.1	392.9	60.81
321 Su	5.61																			
401 SA	5.05	30.9	281.1	529.1	711.5	596.4	260.5	3.36	0.523	0.445	0.473	1.47	38.48	14.14	19.24	144.0	49.82	294.6	581.0	84.94
402 SA	5.13	18.4	162.0	262.0	556.0	764.0	217.8	3.26	0.498	0.270	0.304	1.45	33.92	17.82	18.23	114.2	46.30	227.4	540.9	76.45
403 SA	5.32	25.4	220.2	412.1	707.3	583.1	206.9													
404 SA	5.39	34.0	294.9	388.7	644.2	994.4	198.3													
405 SA	5.46	36.4	332.5	673.4	772.0	503.5	181.2						41.04	44.10	29.07	145.1	33.05	286.5	582.2	93.41
406 SA	5.55	26.8	236.3	503.4	706.1	603.8	223.3						30.01	13.65	19.09	110.3	42.40	213.6	468.0	80.07
407 SA	5.72	29.9	258.6	532.5	676.9	680.0	221.3						32.52	21.48	21.72	118.8	34.15	225.5	547.3	81.03
408 SA	5.74	42.6	369.4	630.9	1011.2	654.8	330.2													
409 SA	5.81	29.4	249.7	468.9	719.3	829.8	198.1						29.63	18.10	26.59	112.1	50.57	198.6	446.4	69.04
410 SA	5.90	40.8	366.2	626.9	757.0	910.4	290.0	3.72	0.716	0.549	0.576	1.95	45.36	25.90	26.18	157.8	51.03	321.5	651.4	104.3
411 SA	5.95	26.0	215.2	492.3	686.9	810.0	213.6	3.14	0.480	0.321	0.300	1.43	32.76	15.37	20.86	113.4	50.48	223.2	470.6	71.88
412 SA	6.01	32.0	277.6	568.1	850.7	584.4	183.3													
413 SA	6.18	50.2	435.4	677.1	982.5	1043.4	342.1													
414 SA	6.26	26.5	226.8	443.3	755.4	474.3	241.7						30.69	17.05	18.38	109.6	36.26	229.1	485.3	69.15
415 SA	6.31	33.5	290.7	449.4	932.6	984.3	262.4													
416 SA	6.47	29.1	252.3	429.0	816.5	925.9	246.6													
417 SA	6.56	47.0	408.2	784.5	1120.2	744.0	245.4													
418 SA	6.69	39.2	340.1	714.0	981.3	592.5	310.3													
419 SA	6.71	39.3	322.3	501.1	674.7	987.1	286.0						26.09	12.38	17.82	99.35	46.99	194.2	416.9	63.70
420 SA	6.87	35.4	305.7	637.9	931.5	695.3	260.3	3.32	0.434	0.342	0.481	1.71	35.19	18.97	24.30	130.0	53.42	243.6	507.1	85.13
421 SA	5.71	25.5	217.1	444.2	842.5	683.2	249.1	3.13	0.362	0.240	0.261	1.37	32.05	19.15	20.49	111.6	37.18	233.0	492.7	76.75

Treatment No		FW	LA	DW leaf	DW N	DW P	DW O	DW Root	%N leaf	%N N	%N P	%N O	%N Root
501	C2	5.05	19.0	149.4	431.4	824.8	1030.5	102.7					
502	C2	5.16	10.9	82.3	173.8	439.9	1125.5	188.5	2.22	0.427	0.237	0.231	1.21
503	C2	5.27	8.1	65.7	313.1	822.7	767.0	154.2					
504	C2	5.36	34.0	266.7	833.4	1151.1	1290.9	216.4					
505	C2	5.46	11.0	93.0	268.6	577.6	927.5	164.9	2.22	0.343	0.255	0.252	1.38
506	C2	5.53	12.3	97.9	349.3	694.3	844.8	185.5					
507	C2	5.69	19.2	156.0	490.0	996.8	1040.1	148.1					
508	C2	5.78	16.8	137.8	422.8	854.2	1223.2	176.5					
509	C2	5.80	12.8	101.1	349.1	841.2	900.2	158.3	2.25	0.323	0.196	0.265	1.16
510	C2	5.81	10.8	89.1	268.6	750.3	995.3	189.7					
511	C2	5.99	11.3	95.1	370.8	918.7	925.1	122.3	2.20	0.368	0.206	0.268	1.20
512	C2	6.02	11.4	96.2	312.3	862.7	865.4	193.0					
513	C2	6.07	16.6	127.5	443.3	827.9	1253.2	160.6	2.39	0.312	0.183	0.250	1.39
514	C2	6.23	14.1	118.2	352.1	832.6	1448.5	202.4					
515	C2	6.35	14.4	111.0	306.1	773.9	1404.1	191.7					
516	C2	6.52	32.1	236.3	591.5	1115.7	1524.5	187.8					
517	C2	6.54	17.2	133.4	271.5	720.7	1317.5	274.9					
518	C2	6.61	28.0	216.5	694.6	1152.7	1437.8	153.3					
519	C2	6.79	19.3	155.1	418.5	921.2	1503.2	233.4					
520	C2	6.85	20.5	158.7	591.1	1113.3	1049.7	205.3					
521	C2	5.76	15.2	121.8	329.3	672.4	994.5	182.7					
522	C2	5.04	12.1	93.3	262.4	569.9	1109.3	95.3	2.27	0.360	0.225	0.281	1.38
601	Wi	5.04	7.8	67.9	196.0	507.9	881.0	168.0					
602	Wi	5.13	9.7	77.3	253.4	636.7	864.1	156.3	2.51	0.548	0.288	0.311	1.22
603	Wi	5.17	17.8	150.6	523.9	975.5	874.0	76.4					
604	Wi	5.40	9.1	73.5	245.6	617.9	934.9	158.3					
605	Wi	5.43	8.9	67.9	220.8	570.8	983.0	109.9					
606	Wi	5.58	22.5	237.0	667.9	1169.5	1204.5	192.3					
607	Wi	5.67	18.1	136.2	373.5	684.8	1259.9	138.1					
608	Wi	5.76	11.5	91.3	265.0	639.8	1184.3	139.3	2.29	0.625	0.300	0.307	1.28
609	Wi	5.83	17.6	138.4	458.4	842.5	1104.5	190.3					
610	Wi	5.88	12.8	101.6	380.4	880.6	968.8	150.3	2.52	0.736	0.365	0.331	1.66
611	Wi	6.00	30.9	249.7	844.1	1225.7	1118.2	252.5					
612	Wi	6.06	14.1	106.6	292.1	756.4	959.6	111.3					
613	Wi	6.08	12.9	109.9	359.9	699.2	1136.1	152.6					
614	Wi	6.20	16.6	141.0	508.4	979.7	874.1	154.2	2.47	0.530	0.330	0.360	1.51
615	Wi	6.35	17.7	133.2	469.7	909.4	1270.1	186.6					
616	Wi	6.38	30.4	239.9	707.9	1084.0	1353.8	271.7					
617	Wi	6.58	16.7	129.3	558.6	1124.2	912.0	164.9					
618	Wi	6.70	19.0	155.8	603.9	1156.9	1070.8	200.5	2.42	0.437	0.293	0.333	1.25
619	Wi	6.74	12.7	97.1	484.6	807.9	926.7	161.0					
620	Wi	6.90	20.6	166.4	564.4	907.8	1411.4	196.1					
621	Wi	5.79	14.0	112.2	305.0	699.4	1445.0	117.7	2.61	0.577	0.305	0.315	1.36
622	Wi	6.32	17.5	126.0	371.6	842.9	1168.4	147.0					
701	SW	5.02	21.7	196.0	1061.0	1034.9	426.0	128.6					
702	SW	5.11	27.4	240.9	1147.3	1191.6	1000.0	211.4					
703	SW	5.21	36.2	314.4	1461.0	1154.4	1205.9	218.4	2.85	0.444	0.354	0.422	1.75
704	SW	5.41	24.4	207.5	947.3	829.8	943.8	229.7	2.72	0.372	0.370	0.355	1.37
705	SW	5.46	25.7	226.5	901.0	922.9	1062.4	136.1					
706	SW	5.60	19.0	148.9	649.5	993.0	791.1	139.7					
707	SW	5.69	27.2	216.4	795.8	1182.7	1015.3	204.3					
708	SW	5.76	31.6	292.8	1457.2	1347.6	576.1	199.5	2.59	0.354	0.292	0.491	1.53
709	SW	5.84	35.4	282.8	965.1	1213.9	1171.9	161.6					
710	SW	5.90			1090.8	1262.1	1188.7	257.3					
711	SW	5.99	36.1	288.6	1267.6	1514.6	911.2	231.7					
712	SW	6.04	48.9	397.8	1728.1	1348.6	1409.0	257.5					
713	SW	6.17	35.1	292.7	1135.2	1331.1	1247.3	164.7					
714	SW	6.27	39.4	318.8	1268.5	1481.1	1342.3	230.1					
715	SW	6.32	30.1	266.0	975.5	1111.7	1185.4	202.3	2.62	0.352	0.265	0.368	1.47
716	SW	6.53	36.0	338.0	1598.8	1403.8	1225.6	253.6					
717	SW	6.57	29.8	244.2	1143.7	1314.7	1025.6	285.1	2.65	0.376	0.260	0.362	1.40
718	SW	6.63	44.0	344.9	1367.5	1411.9	1610.0	206.4					
719	SW	6.72	40.6	332.7	1443.6	1426.2	1477.3	329.6	2.59	0.385	0.277	0.424	1.63
720	SW	6.85	39.6	390.3	1872.9	1393.5	869.5	286.7					
721	SW	5.40	13.2	101.5	361.9	627.5	1036.5	182.0					
722	SW	6.14	43.2	346.5	1491.8	1444.2	1505.9	182.3					

NL: number of leaves

Treatment No		NL 1 Oct	NL 7 Oct	NL 16 Oct	NL 21 Oct	NL 28 Oct	NL 4 Nov	NL 11 Nov	NL 18 Nov	NL 2 Dec	NL 16 Dec	NL 28 Dec
1	C0											
2	C0											
3	C0											
4	C0											
5	C0											
6	C0											
7	C0											
8	C0											
9	C0											
10	C0											
11	C0											
12	C0											
13	C0											
14	C0											
15	C0											
16	C0											
17	C0											
18	C0											
19	C0											
20	C0											
101	C1	0	2	2	2	2	2	2	2	2	2	2
102	C1	2	3	3	3	3	3	3	3	3	3	3
103	C1	0	3	3	3	3	3	3	3	3	3	3
104	C1	0	2	2	2	3	3	3	3	3	3	3
105	C1	0	2	2	2	2	2	2	2	2	2	2
106	C1	0	0	2	2	2	2	2	2	2	2	2
107	C1	0	3	3	3	3	3	3	3	3	3	3
108	C1	2	4	4	4	4	4	4	4	4	4	4
109	C1	0	0	3	3	3	3	3	3	3	3	3
110	C1	0	2	2	2	2	2	2	2	2	2	2
111	C1	0	2	2	2	2	2	2	2	2	2	2
112	C1	0	0	2	4	5	5	5	5	5	5	5
113	C1	0	3	4	4	4	4	4	4	4	4	4
114	C1	0	2	2	2	2	2	2	2	2	2	2
115	C1	0	3	5	6	6	6	6	6	6	6	6
116	C1	0	3	3	3	3	3	3	3	3	3	3
117	C1	0	2	3	4	4	4	4	4	4	4	4
118	C1	0	3	3	3	3	3	3	3	3	3	3
119	C1	0	3	4	4	4	4	4	4	4	4	4
120	C1	2	3	3	3	3	3	3	3	3	3	3
121	C1	0	2	2	2	2	2	2	2	2	2	2

Treatment No		NL 1 Oct	NL 7 Oct	NL 16 Oct	NL 21 Oct	NL 28 Oct	NL 4 Nov	NL 11 Nov	NL 18 Nov	NL 2 Dec	NL 16 Dec	NL 28 Dec
201	Au	0	3	3	3	3	3	3	3	4	4	4
202	Au	2	3	4	4	4	4	4	4	4	4	4
203	Au	0	2	2	2	2	2	2	2	2	2	2
204	Au	2	3	3	3	3	3	3	3	3	3	3
205	Au	0	0	2	2	2	2	3	3	3	3	3
206	Au	0	0	2	2	2	2	2	2	2	2	2
207	Au	0	2	2	2	2	2	3	3	3	3	3
208	Au	0	2	2	2	2	2	2	3	3	3	3
209	Au	2	3	5	5	5	5	6	6	6	6	6
210	Au	0	3	3	3	3	3	3	3	3	3	3
211	Au	1	3	3	3	3	3	3	3	4	4	4
212	Au	0	2	2	2	2	3	3	3	3	3	3
213	Au	0	3	3	3	3	3	3	3	3	3	4
214	Au	0	2	2	2	2	2	2	2	2	2	3
215	Au	0	2	3	3	3	3	3	3	3	3	3
216	Au	0	2	4	4	4	4	4	4	4	4	4
217	Au	0	3	3	3	3	3	3	3	3	3	3
218	Au	1	3	3	3	3	3	3	3	3	3	3
219	Au	0	2	5	5	5	5	5	5	5	5	5
220	Au	0	5	5	5	5	5	5	5	5	5	5
221	Au	0	3	4	4	4	4	4	4	4	4	4
301	Su	0	4	6	6	6	6	6	6	6	6	6
302	Su	0	3	4	4	4	4	4	4	4	4	4
303	Su	2	3	4	4	4	4	4	4	4	4	4
304	Su	0	3	5	5	5	5	5	5	5	5	5
305	Su	0	2	3	3	3	3	3	3	3	3	3
306	Su	3	5	5	5	6	6	6	6	6	6	6
307	Su	3	5	5	6	6	6	6	6	6	6	6
308	Su	0	3	4	4	4	4	4	4	4	4	4
309	Su	0	3	4	4	4	4	4	4	4	4	4
310	Su	0	3	3	3	3	3	3	3	3	3	3
311	Su	2	4	4	4	4	4	4	4	4	4	4
312	Su	0	4	4	4	4	4	4	4	4	4	4
313	Su	0	3	3	3	3	4	4	4	4	4	4
314	Su	2	5	5	5	5	5	5	5	5	5	5
315	Su	3	4	4	4	4	4	4	4	4	4	4
316	Su	0	5	5	5	5	5	5	5	5	5	5
317	Su	0	3	3	3	3	3	3	4	4	4	4
318	Su	0	2	3	3	3	3	3	3	3	3	3
319	Su	4	5	5	5	5	5	5	5	5	5	5
320	Su	0	3	4	4	4	4	4	4	4	4	4
321	Su	3	6									
401	SA	2	3	4	4	4	4	4	4	4	4	4
402	SA	2	3	3	3	3	3	3	3	3	3	3
403	SA	0	2	5	5	5	5	5	5	5	5	5
404	SA	1	5	5	5	5	5	5	5	5	5	5
405	SA	2	3	4	4	4	4	4	4	4	4	4
406	SA	0	3	4	4	4	4	4	4	4	4	4
407	SA	0	3	4	4	4	4	4	4	4	4	4
408	SA	2	4	6	6	6	6	6	6	6	6	6
409	SA	0	3	3	4	4	4	4	4	4	4	4
410	SA	3	4	4	4	4	4	4	4	4	4	4
411	SA	0	3	3	3	3	3	3	3	3	4	4
412	SA	0	3	5	5	5	5	5	5	5	5	5
413	SA	3	5	5	5	5	5	5	5	5	5	5
414	SA	0	3	4	4	4	4	4	4	4	4	4
415	SA	2	3	5	5	5	5	5	5	5	5	5
416	SA	2	5	5	5	5	5	5	5	5	5	5
417	SA	0	5	5	5	5	5	6	6	6	6	6
418	SA	0	5	5	5	5	5	5	6	6	6	6
419	SA	0	3	4	4	4	4	4	4	4	4	4
420	SA	0	4	4	4	4	4	4	4	4	4	4
421	SA	0	3	3	3	3	3	3	3	3	3	3

Treatment No	NL 1 Oct	NL 7 Oct	NL 16 Oct	NL 21 Oct	NL 28 Oct	NL 4 Nov	NL 11 Nov	NL 18 Nov	NL 2 Dec	NL 16 Dec	NL 28 Dec	NL 21 Jan	NL 4 Feb	NL 18 Feb	NL 4 Mar	NL 17 Mar
501 C2	0	3	4	4	4	4	4	4	4	4	4	4	4	4	4	4
502 C2	0	2	2	2	2	2	2	2	2	2	2	2	2	2	2	2
503 C2	0	0	2	2	2	2	2	2	2	2	2	2	2	2	2	2
504 C2	4	4	5	5	5	5	5	5	5	5	5	5	5	5	5	5
505 C2	0	2	2	2	2	3	3	3	3	3	3	3	3	3	3	3
506 C2	0	2	3	3	3	3	3	3	3	3	3	3	3	3	3	3
507 C2	0	2	3	3	3	3	4	4	4	4	4	4	4	4	4	4
508 C2	0	2	2	3	3	3	3	3	3	3	3	3	3	3	3	3
509 C2	0	2	3	3	3	3	3	3	3	3	3	3	3	3	3	3
510 C2	0	0	2	2	2	2	2	2	2	2	2	2	2	2	2	2
511 C2	0	0	2	2	2	2	2	2	2	2	2	2	2	2	2	2
512 C2	0	2	2	2	2	2	2	2	2	2	2	2	2	2	2	2
513 C2	0	2	3	3	3	3	3	3	3	3	3	3	3	3	3	3
514 C2	2	2	2	2	2	2	2	2	2	2	2	2	2	2	2	2
515 C2	0	1	2	2	3	3	3	3	3	3	3	3	3	3	3	3
516 C2	1	4	5	5	5	5	5	5	5	5	5	5	5	5	5	5
517 C2	0	2	3	3	3	3	3	3	3	3	3	3	3	3	3	3
518 C2	1	4	4	4	4	4	4	4	4	4	4	4	4	4	4	4
519 C2	0	4	4	4	4	4	4	4	4	4	4	4	4	4	4	4
520 C2	0	0	2	4	4	5	5	5	5	5	5	5	5	5	5	5
521 C2	0	2	3	3	3	3	3	3	3	3	3	3	3	3	3	3
522 C2	0	2	2	2	2	2	2	2	2	2	2	2	2	2	2	2
601 Wi	0	0	2	2	2	2	2	2	2	2	2	2	2	2	2	2
602 Wi	0	1	2	2	2	2	2	2	2	2	2	2	2	2	2	2
603 Wi	0	3	3	3	3	3	3	3	3	3	3	3	3	3	3	3
604 Wi	0	2	2	2	2	2	2	2	2	2	2	2	2	2	2	2
605 Wi	0	0	2	2	2	2	2	2	2	2	2	2	2	2	2	2
606 Wi	3	4	4	4	4	4	4	4	4	4	4	4	4	4	4	4
607 Wi	0	0	4	4	4	4	4	4	4	4	4	4	4	4	4	4
608 Wi	0	2	2	2	2	2	2	2	2	2	2	2	2	2	2	2
609 Wi	0	3	4	4	4	4	4	4	4	4	4	4	4	4	4	4
610 Wi	0	2	2	2	2	2	2	2	2	2	2	2	2	2	2	2
611 Wi	0	4	5	5	5	5	5	5	5	5	5	5	5	5	5	5
612 Wi	0	1	3	3	3	3	3	3	3	3	3	3	3	3	3	3
613 Wi	0	2	2	3	3	3	3	3	3	3	3	3	3	3	3	3
614 Wi	0	2	2	2	2	2	2	3	3	3	3	3	3	3	3	3
615 Wi	0	2	3	3	3	3	3	3	3	3	3	3	3	3	3	3
616 Wi	0	3	5	5	5	5	5	5	5	5	5	5	5	5	5	5
617 Wi	0	3	3	3	3	3	3	3	3	3	3	3	3	3	3	3
618 Wi	0	2	3	3	3	3	3	3	3	3	3	3	3	3	3	3
619 Wi	0	0	2	2	2	2	2	2	3	3	3	3	3	3	3	3
620 Wi	0	3	3	4	5	5	5	5	5	5	5	5	5	5	5	5
621 Wi	0	2	2	2	2	2	2	2	2	2	2	2	2	2	2	2
622 Wi	0	2	3	3	3	3	4	4	4	4	4	4	4	4	4	4
701 SW	0	3	3	3	3	3	3	3	3	3	3	3	3	3	3	3
702 SW	2	3	3	3	3	3	3	3	3	3	3	3	3	3	3	3
703 SW	0	3	4	4	4	4	4	4	4	4	4	4	4	4	4	4
704 SW	0	3	3	3	3	3	3	4	4	4	4	4	4	4	4	4
705 SW	0	3	3	3	3	3	3	3	3	4	4	4	4	4	4	4
706 SW	0	1	3	3	3	3	3	3	3	3	3	3	3	3	3	3
707 SW	2	5	5	5	5	5	5	5	5	5	5	5	5	5	5	5
708 SW	0	3	4	4	4	4	4	4	4	4	4	4	4	4	4	4
709 SW	2	5	5	5	5	5	5	5	5	5	5	5	5	5	5	5
710 SW	2	3	3	3	4	4	4	4	4	4	4	4	4	4	4	4
711 SW	1	4	5	5	5	5	5	5	5	5	5	5	5	5	5	5
712 SW	2	5	6	6	6	6	6	6	7	7	7	7	7	7	7	7
713 SW	0	5	5	5	5	5	5	5	5	5	5	5	5	5	5	5
714 SW	2	5	5	5	5	5	5	5	5	5	5	5	5	5	5	5
715 SW	0	3	4	4	4	4	4	4	4	4	4	4	4	4	4	4
716 SW	2	4	4	4	4	4	4	4	4	4	4	4	4	4	4	4
717 SW	2	3	3	3	3	4	4	4	4	4	4	4	4	4	4	4
718 SW	2	4	4	4	4	4	4	4	4	4	4	4	4	4	4	4
719 SW	3	4	4	4	4	4	4	4	4	4	4	4	4	4	4	4
720 SW	3	4	4	4	4	4	4	5	5	5	5	5	5	5	5	5
721 SW	0	2	2	3	3	3	3	3	3	3	3	3	3	3	3	3
722 SW	3	3	4	4	4	4	4	4	4	4	4	4	4	4	4	4

LL: total leaf length (mm)

Treatment No		LL 1 Oct	LL 7 Oct	LL 16 Oct	LL 21 Oct	LL 28 Oct	LL 4 Nov	LL 11 Nov	LL 18 Nov	LL 2 Dec	LL 16 Dec	LL 28 Dec
1	C0											
2	C0											
3	C0											
4	C0											
5	C0											
6	C0											
7	C0											
8	C0											
9	C0											
10	C0											
11	C0											
12	C0											
13	C0											
14	C0											
15	C0											
16	C0											
17	C0											
18	C0											
19	C0											
20	C0											
101	C1	0	11	77	106	155	161	177	185	203	229	233
102	C1	14	73	181	215	252	291	316	325	359	378	382
103	C1	0	69	191	228	258	303	326	335	371	400	401
104	C1	0	22	82	104	134	160	182	194	223	250	263
105	C1	0	21	88	116	142	165	186	196	215	231	235
106	C1	0	0	31	54	79	102	114	124	151	167	174
107	C1	0	18	113	148	187	221	244	251	290	318	334
108	C1	2	118	298	362	419	467	492	508	565	602	618
109	C1	0	0	57	96	136	172	192	209	250	272	280
110	C1	0	3	52	81	104	127	146	151	166	184	187
111	C1	0	23	82	105	129	154	162	178	197	215	220
112	C1	0	0	93	166	262	345	383	419	504	551	576
113	C1	0	69	210	265	333	381	401	412	463	484	501
114	C1	0	8	65	94	125	150	167	176	194	210	232
115	C1	0	42	189	282	374	460	502	519	606	636	657
116	C1	0	31	135	169	205	238	258	275	291	314	322
117	C1	0	32	138	180	222	270	298	311	351	376	389
118	C1	0	43	153	195	237	270	284	298	358	381	389
119	C1	0	38	148	193	255	291	310	341	392	420	436
120	C1	13	81	198	241	283	318	340	359	398	422	432
121	C1	0	10	79	104	135	158	176	187	202	217	221

Treatment		LL	LL	LL	LL	LL	LL	LL	LL	LL	LL	LL
No		1 Oct	7 Oct	16 Oct	21 Oct	28 Oct	4 Nov	11 Nov	18 Nov	2 Dec	16 Dec	28 Dec
201	Au	0	44	166	235	300	356	398	424	522	590	618
202	Au	8	96	247	325	404	485	524	553	624	685	705
203	Au	0	10	78	110	146	172	189	210	239	262	272
204	Au	12	73	220	292	362	429	456	471	531	540	543
205	Au	0	0	76	104	142	180	209	232	291	331	357
206	Au	0	0	48	79	111	137	158	170	199	213	224
207	Au	0	33	107	136	164	196	222	242	310	352	369
208	Au	0	54	130	166	202	236	256	280	349	404	414
209	Au	2	73	219	296	376	446	479	503	605	655	635
210	Au	0	27	119	155	199	240	148	184	241	276	290
211	Au	1	82	234	295	365	430	452	475	556	601	628
212	Au	0	12	74	104	128	166	191	213	260	292	321
213	Au	0	69	188	236	290	337	372	390	449	491	505
214	Au	0	13	80	107	138	170	195	209	239	263	276
215	Au	0	18	149	220	288	344	376	403	465	521	534
216	Au	0	31	175	228	294	352	382	400	463	510	529
217	Au	0	51	167	220	272	318	338	362	420	454	470
218	Au	1	60	176	214	250	287	318	342	362	388	397
219	Au	0	14	164	265	350	418	457	486	536	596	621
220	Au	0	60	240	321	402	479	519	544	631	700	710
221	Au	0	53	201	246	301	352	382	399	457	483	504
301	Su	0	85	311	416	514	577	613	637	723	754	776
302	Su	0	75	215	284	340	396	418	442	520	555	582
303	Su	10	123	319	401	468	518	542	569	611	663	683
304	Su	0	70	290	375	457	514	549	579	651	682	697
305	Su	0	18	144	218	245	291	310	330	372	391	393
306	Su	27	180	419	503	577	653	691	728	794	843	867
307	Su	16	167	393	473	560	637	688	702	782	841	851
308	Su	0	11	136	217	281	337	365	392	461	507	515
309	Su	0	90	283	381	470	535	568	591	668	715	728
310	Su	0	106	275	336	374	427	454	468	516	554	564
311	Su	4	107	316	395	458	513	542	563	620	671	679
312	Su	0	78	275	352	423	481	517	542	601	663	672
313	Su	0	75	227	285	335	372	404	434	493	548	573
314	Su	24	199	454	524	587	636	613	648	672	712	735
315	Su	9	108	321	405	463	532	565	587	642	671	702
316	Su	0	95	283	375	401	482	508	520	567	588	614
317	Su	0	75	193	241	285	333	358	376	420	445	473
318	Su	0	48	152	201	245	293	312	324	370	363	360
319	Su	29	196	429	504	580	647	681	696	758	783	798
320	Su	0	56	226	314	382	442	474	490	553	601	619
321	Su	12	170									
401	SA	16	111	252	318	371	434	466	496	574	622	638
402	SA	6	69	190	243	277	318	333	350	384	411	415
403	SA	0	26	196	282	366	432	480	505	557	614	622
404	SA	3	115	371	448	522	586	511	551	600	653	673
405	SA	14	134	334	415	489	551	594	611	670	724	746
406	SA	0	36	179	258	326	387	428	449	527	567	593
407	SA	0	80	233	305	369	433	471	494	564	612	626
408	SA	30	137	356	463	548	646	683	721	846	893	922
409	SA	0	43	185	260	338	410	441	471	545	599	617
410	SA	25	135	339	423	495	562	605	632	714	755	799
411	SA	0	68	225	286	334	378	404	422	462	497	515
412	SA	0	56	302	372	445	506	547	573	629	682	693
413	SA	42	199	485	592	701	767	814	839	906	968	1005
414	SA	0	32	187	264	327	379	415	434	520	551	561
415	SA	16	111	312	400	490	542	586	595	661	711	731
416	SA	6	124	307	380	449	506	536	559	621	651	681
417	SA	0	145	405	525	621	684	734	770	877	955	962
418	SA	0	56	290	377	473	543	594	634	718	806	823
419	SA	0	88	272	351	423	501	539	570	669	724	753
420	SA	0	90	279	359	426	488	517	548	633	674	684
421	SA	0	65	220	278	319	369	393	414	470	495	508

Treatment No		LL 1 Oct	LL 7 Oct	LL 16 Oct	LL 21 Oct	LL 28 Oct	LL 4 Nov	LL 11 Nov	LL 18 Nov	LL 2 Dec	LL 16 Dec	LL 28 Dec	LL 21 Jan	LL 4 Feb	LL 18 Feb	LL 4 Mar	LL 17 Mar
501	C2	0	42	163	217	274	323	347	368	413	416	426	420	433	431	447	450
502	C2	0	11	70	95	120	144	158	171	192	204	229	219	224	236	248	264
503	C2	0	0	15	36	62	83	100	109	134	152	157	160	162	174	186	191
504	C2	32	163	349	431	488	549	583	599	654	690	706	707	724	725	739	738
505	C2	0	23	79	99	117	142	162	177	204	223	228	230	247	264	265	267
506	C2	0	13	82	107	142	171	195	210	233	260	263	273	283	294	318	332
507	C2	0	23	127	166	208	239	278	290	349	383	392	404	420	424	442	449
508	C2	0	40	118	153	201	237	263	279	317	317	323	316	320	331	348	364
509	C2	0	2	83	118	158	189	209	222	255	273	276	279	281	302	322	328
510	C2	0	0	53	76	101	127	144	150	180	194	201	203	205	212	225	244
511	C2	0	0	31	65	100	140	159	171	191	206	214	217	229	233	241	246
512	C2	0	29	87	110	132	151	163	174	198	216	218	220	224	233	243	249
513	C2	0	26	117	162	195	239	261	280	314	337	343	343	348	362	368	379
514	C2	8	68	161	189	220	247	259	268	298	310	318	319	327	335	348	322
515	C2	0	6	66	94	145	185	220	233	269	283	293	299	311	322	340	350
516	C2	1	150	291	370	419	489	525	535	578	608	624	632	637	655	694	716
517	C2	0	43	131	175	178	251	270	285	312	332	349	348	361	364	390	399
518	C2	1	115	297	365	409	466	498	518	548	546	552	553	556	562	564	566
519	C2	0	33	144	198	256	291	311	326	354	387	401	409	420	428	449	454
520	C2	0	0	34	104	145	259	299	323	393	428	440	446	449	464	481	497
521	C2	0	41	120	168	209	246	261	279	309	328	330	335	346	348	358	372
522	C2	0	32	102	130	158	183	200	206	224	243	245	249	258	260	274	271
601	Wi	0	0	32	53	75	95	103	116	133	151	159	161	164	175	186	195
602	Wi	0	5	54	84	107	128	144	157	178	194	200	202	205	212	229	234
603	Wi	0	29	135	179	212	262	278	293	325	343	357	361	370	379	391	397
604	Wi	0	11	66	87	105	128	148	154	177	187	193	200	205	214	224	225
605	Wi	0	0	41	66	93	112	127	136	156	169	172	176	187	189	204	221
606	Wi	24	157	324	380	431	466	496	517	541	544	560	567	571	576	612	618
607	Wi	0	0	90	140	195	248	283	299	336	368	377	385	407	416	443	464
608	Wi	0	7	69	99	126	154	168	178	204	213	216	218	226	237	246	249
609	Wi	0	36	136	181	233	280	309	323	355	390	404	406	419	434	431	435
610	Wi	0	6	74	106	133	159	171	185	208	217	227	228	240	246	265	272
611	Wi	0	62	261	329	406	470	504	530	589	626	634	641	672	668	705	732
612	Wi	0	4	79	127	167	206	232	240	273	291	307	306	311	324	330	337
613	Wi	0	7	62	89	124	160	180	203	238	251	261	270	274	279	302	317
614	Wi	0	16	83	112	150	179	192	219	256	286	297	305	315	331	347	361
615	Wi	0	12	98	140	185	225	243	263	297	315	320	325	327	342	367	386
616	Wi	0	60	214	281	360	430	461	491	549	577	597	613	626	637	679	696
617	Wi	0	24	120	156	192	233	262	269	300	319	323	330	330	331	358	355
618	Wi	0	28	123	169	209	252	276	285	320	342	353	354	355	379	398	410
619	Wi	0	0	30	57	93	122	135	149	174	205	210	221	233	251	278	282
620	Wi	0	32	116	171	238	299	327	352	417	448	451	464	482	495	513	508
621	Wi	0	36	103	135	161	180	203	213	235	252	256	256	263	269	286	285
622	Wi	0	8	92	135	176	214	223	244	295	329	336	335	348	357	377	391
701	SW	0	35	160	209	260	306	339	350	387	409	421	430	436	446	465	470
702	SW	8	94	239	287	332	371	391	415	463	493	496	505	524	530	553	573
703	SW	0	71	257	338	396	460	496	523	592	631	649	647	677	695	738	755
704	SW	0	35	169	229	285	332	356	388	455	498	505	496	506	504	495	507
705	SW	0	59	195	245	291	336	360	379	424	467	477	490	505	511	538	542
706	SW	0	2	116	172	224	280	302	317	346	366	372	377	382	400	412	433
707	SW	18	120	281	344	396	437	456	471	510	537	548	556	570	580	593	606
708	SW	0	24	202	292	357	408	447	459	523	566	572	580	587	607	632	645
709	SW	5	143	410	495	576	633	663	675	707	692	690	688	686	686	713	702
710	SW	10	123	250	304	370	425	458	474	523	560	569	575	594	612	653	678
711	SW	1	102	304	384	457	516	553	571	622	649	658	661	676	686	727	736
712	SW	15	139	387	486	586	664	708	739	826	901	902	921	948	963	1049	1075
713	SW	0	71	343	427	517	588	634	655	699	724	732	701	690	656	656	690
714	SW	12	172	380	453	518	571	615	627	676	714	723	739	749	760	781	782
715	SW	0	40	224	301	366	424	446	466	518	555	573	582	601	616	650	692
716	SW	6	121	304	385	430	487	518	547	600	631	648	655	682	687	726	735
717	SW	2	76	214	270	333	361	395	409	465	510	524	533	544	558	584	594
718	SW	2	112	352	443	513	581	604	625	683	715	725	732	744	746	755	816
719	SW	60	197	403	474	525	584	609	620	683	710	726	736	751	710	738	752
720	SW	25	143	337	405	461	506	548	573	635	696	618	625	655	676	729	720
721	SW	0	30	88	118	155	178	197	206	235	254	272	277	283	288	308	313
722	SW	32	165	396	470	540	598	625	649	692	720	736	741	752	769	797	791
