# Supplementary material for: Functional differences in seasonally absorbed nitrogen in a winter-green perennial herb
Source: R Soc Open Sci. 2020 Jan 29;7(1):190034. doi: 10.1098/rsos.190034 (PMC7029918; doi:10.1098/rsos.190034)
Supplement: Data for photosynthetic capacity [file rsos190034supp3.pdf]

Nishitani et al. "Functional differences in seasonally absorbed nitrogen in a winter-green perennial herb" Royal Society Open Science

Photosynthesis rate in relation to partial pressure of intercellular CO_2

Ca: partial pressure of ambient CO_2 (Pa)
Ci: partial pressure of intercellular CO_2 (Pa)
PhA: area-based photosynthesis rate (μmol CO_2 m^{-2} s^{-1})
LMA: leaf mass per area (mg cm^{-2})

Control C1 Leaf N concentration 0.160 mol N m^{-2}

	No 101		No 116		No 105		No 110		No 102		No 118		No 111		No 120	
Ca	Ci	PhA	Ci	PhA	Ci	PhA	Ci	PhA	Ci	PhA	Ci	PhA	Ci	PhA	Ci	PhA
0	1.3	-3.82	1.3	-3.82	1.3	-3.78	1.4	-3.83	1.2	-3.63	0.9	-2.81	1.2	-4.00	1.4	-3.78
10	8.7	3.23	8.9	2.70	8.5	3.64	8.6	3.22	8.5	3.63	8.6	3.40	8.8	3.26	8.6	3.52
20	16.2	9.55	16.0	9.98	15.8	10.44	15.8	10.13	15.8	10.23	16.0	10.43	16.2	10.33	15.5	11.52
29	23.3	17.27	23.7	16.40	23.0	17.42	22.6	17.30	23.1	17.12	23.2	17.02	23.5	17.12	23.3	17.61
36	28.9	20.91	29.0	20.67	28.2	21.42	28.1	21.09	28.4	20.93	28.5	21.28	29.2	21.02	28.5	21.96
119	104.4	39.55	105.4	37.93	102.3	41.91	102.3	39.78	104.4	37.21	105.4	35.79	104.6	38.60	105.4	38.70
LMA	8.15		8.18		8.18		8.06		8.20		8.14		8.15		7.93	

Treatment Su Leaf N concentration 0.185 mol N m^{-2}

	No 313		No 303		No 302		No 315		No 308		No 311		No 320		No 312	
Ca	Ci	PhA	Ci	PhA	Ci	PhA	Ci	PhA	Ci	PhA	Ci	PhA	Ci	PhA	Ci	PhA
0	1.7	-3.80	1.1	-3.38	1.5	-4.04	1.2	-3.20	1.3	-4.00	1.3	-3.48	1.4	-3.76	1.3	-3.59
10	8.3	3.33	8.3	4.00	8.1	4.22	8.3	3.81	8.6	3.71	8.5	3.68	8.6	3.01	7.9	4.74
20	15.0	10.27	14.7	12.04	15.2	11.83	15.2	11.43	15.6	11.45	15.7	10.96	15.3	10.30	14.9	12.16
29	22.3	16.16	21.7	19.59	21.8	19.78	22.3	18.29	22.7	18.69	22.8	17.40	22.3	16.79	21.8	19.38
36	27.4	20.24	27.1	23.71	26.2	24.39	27.4	22.86	27.6	23.49	27.5	22.20	27.2	21.39	26.9	23.92
119	102.3	37.25	103.4	41.65	99.9	45.16	102.3	41.14	103.4	41.68	105.4	36.08	101.6	38.81	102.3	40.41
LMA	8.13		8.64		8.75		8.16		8.60		8.40		8.08		8.53	

Treatment Au Leaf N concentration 0.204 mol N m^{-2}

	No 208		No 211		No 207		No 215		No 205		No 217		No 212		No 213	
Ca	Ci	PhA	Ci	PhA	Ci	PhA	Ci	PhA	Ci	PhA	Ci	PhA	Ci	PhA	Ci	PhA
0	1.4	-3.67	1.5	-3.92	1.6	-4.26	1.4	-3.79	1.8	-4.10	1.2	-3.79	1.4	-3.91	1.2	-3.66
10	8.0	4.74	8.3	3.79	8.4	3.74	8.0	4.33	8.2	4.05	8.3	4.38	8.4	3.57	8.3	4.43
20	14.7	12.70	15.0	11.22	15.2	11.43	14.6	12.37	14.9	11.71	15.5	12.29	15.3	11.28	15.2	12.26
29	21.8	19.59	21.7	18.97	22.4	18.55	21.5	19.46	21.1	19.28	22.8	19.41	22.4	17.96	22.5	19.14
36	26.8	24.45	27.0	23.09	27.0	23.08	26.5	24.08	26.6	23.37	28.0	24.17	27.4	22.64	27.5	24.00
119	99.3	48.66	102.0	42.06	97.8	49.67	100.5	44.12	99.9	46.51	103.1	46.32	100.6	44.68	101.3	46.45
LMA	8.51		8.57				8.77		8.88		8.65		8.87		8.84	

Treatment SA Leaf N concentration 0.207 mol N m^{-2}

	No 409		No 405		No 406		No 419		No 407		No 420		No 401		No 410	
Ca	Ci	PhA	Ci	PhA	Ci	PhA	Ci	PhA	Ci	PhA	Ci	PhA	Ci	PhA	Ci	PhA
0	1.6	-3.99	1.6	-4.04	1.7	-3.91	1.4	-3.38	1.4	-3.83	1.1	-3.69	1.4	-4.33	1.2	-3.46
10	8.4	3.54	8.0	4.60	8.3	3.62	8.2	4.04	8.4	3.88	8.5	4.31	8.3	4.25	8.0	4.96
20	14.9	11.12	14.9	12.80	14.8	11.36	15.1	11.12	15.3	11.83	15.7	12.35	15.3	12.37	14.6	13.46
29	21.9	18.32	21.8	20.40	20.8	19.23	22.0	18.46	22.4	18.88	22.9	19.53	22.5	19.55	21.6	21.04
36	26.9	22.95	26.7	25.80	26.3	23.19	26.9	22.85	27.6	23.25	28.1	24.51	27.7	23.63	25.8	25.77
119	101.7	43.37	102.2	44.00	99.0	46.38	102.3	40.00	100.7	47.08	105.2	42.35	102.3	44.95	102.3	45.77
LMA	8.50		9.13		8.82		8.20		8.64		8.63		9.09		8.98	
