# Supplementary material for: Functional differences in seasonally absorbed nitrogen in a winter-green perennial herb
Source: R Soc Open Sci. 2020 Jan 29;7(1):190034. doi: 10.1098/rsos.190034 (PMC7029918; doi:10.1098/rsos.190034)
Supplement: Data for chlorophyll a (Chla) fluorescence yield [file rsos190034supp4.pdf]

Nishitani et al. "Functional differences in seasonally absorbed nitrogen in a winter-green perennial herb" Royal Society Open Science

Chl*a* fluorescence yield

Data obtained at an internal temperature of t °C (F_t) was converted to that at 20 °C (F_{20}) by assuming that a 1°C increase results in a 1% decrease in the measuring light intensity: $F_{20} = F_t\,(1\text{-}0.01)^{20\text{-}t}$

Measurement of F_o and F_m in January

at nearly unstressed state					after a sunny day			
No.	Treatment	t (°C)	$F_{o\,20}$	$F_{m\,20}$	Night temperature	t (°C)	$F_{o\,20}$	$F_{m\,20}$
102	C1	21	230	1031	5 °C	21	238	844
105	C1	22	240	950	5 °C	22	246	749
114	C1	23	257	1139	5 °C	23	245	802
116	C1	24	240	1136	5 °C	24	242	931
118	C1	25	249	1205	5 °C	25	244	930
121	C1	25	240	1123	5 °C	25	227	919
101	C1	23	254	1141	-8 °C (min)	23	255	811
104	C1	23	234	1035	-8 °C (min)	23	234	784
107	C1	24	251	1236	-8 °C (min)	24	255	916
110	C1	25	251	1213	-8 °C (min)	25	246	896
111	C1	25	253	1085	-8 °C (min)	25	252	774
120	C1	26	263	1251	-8 °C (min)	26	274	906
302	Su	21	255	1180	5 °C	21	242	868
305	Su	22	246	1152	5 °C	22	243	910
309	Su	24	270	1243	5 °C	24	264	965
311	Su	24	279	1288	5 °C	24	276	1037
315	Su	25	286	1393	5 °C	25	283	1018
320	Su	25	283	1365	5 °C	25	276	1075
303	Su	23	275	1254	-8 °C (min)	23	295	938
308	Su	23	256	1146	-8 °C (min)	23	266	897
310	Su	25	299	1407	-8 °C (min)	25	286	951
312	Su	25	269	1226	-8 °C (min)	25	273	956
313	Su	26	288	1300	-8 °C (min)	25	286	1003
317	Su	26	290	1260	-8 °C (min)	26	291	1035
205	Au	21	236	1156	5 °C	21	233	895
206	Au	22	242	1193	5 °C	23	237	931
211	Au	23	267	1262	5 °C	24	259	956
214	Au	24	251	1047	5 °C	24	245	866
215	Au	25	267	1248	5 °C	25	263	939
217	Au	25	257	1217	5 °C	25	249	997
203	Au	23	252	1203	-8 °C (min)	23	271	927
207	Au	23	264	1246	-8 °C (min)	23	277	909
208	Au	25	268	1290	-8 °C (min)	24	274	913
212	Au	25	260	1321	-8 °C (min)	24	263	980
213	Au	26	264	1216	-8 °C (min)	26	278	917
218	Au	26	275	1306	-8 °C (min)	26	276	1047
402	SA	22	245	1257	5 °C	22	255	966
405	SA	23	284	1139	5 °C	23	278	948
406	SA	24	256	1284	5 °C	24	251	946
410	SA	24	275	1426	5 °C	24	274	1123
414	SA	25	266	1198	5 °C	25	259	953
420	SA	25	285	1313	5 °C	25	284	1060
401	SA	23	268	1314	-8 °C (min)	23	293	913
407	SA	23	261	1320	-8 °C (min)	23	271	1006
409	SA	24	267	1127	-8 °C (min)	25	271	859
411	SA	25	280	1314	-8 °C (min)	25	299	977
419	SA	25	285	1444	-8 °C (min)	26	310	1016
421	SA	26	277	1252	-8 °C (min)	26	287	1014

Measurement of F and F_m' in January

Data obtained when PPFD >1100 μmol m^{-2} s^{-1} are shown in red

No.	Treatment	Night temperature	PPFD (μmol m^{-2} s^{-1})			t (°C)			F_{20}			$F_m'_{20}$		
			1	2	3	1	2	3	1	2	3	1	2	3
102	C1	5 °C	1080	1058	1243	17	25	29	185	193	188	228	253	244
105	C1	5 °C	1006	1035	1278	18	26	29	193	216	212	240	287	264
114	C1	5 °C	1066	1048	1219	19	27	30	223	259	250	302	373	346
116	C1	5 °C	1086	1004	1212	20	28	30	236	235	212	320	336	274
118	C1	5 °C	1065	1145	1272	23	28	30	211	231	215	290	300	276
121	C1	5 °C	897	1152	1198	23	28	30	222	212	208	318	287	276
101	C1	-8 °C (min)	1031	1065	1263	18	27	29	163	185	188	180	225	231
104	C1	-8 °C (min)	1061	1093	1289	19	27	29	140	175	172	155	226	211
107	C1	-8 °C (min)	1101	1018	1245	21	28	30	157	187	186	183	237	234
110	C1	-8 °C (min)	1060	932	1234	22	28	30	152	196	209	177	259	273
111	C1	-8 °C (min)	904	1176	1202	24	28	31	189	205	203	238	258	254
120	C1	-8 °C (min)	997	1213	1228	25	28	31	190	191	201	257	248	264
302	Su	5 °C	1023	1039	1277	17	26	29	248	262	247	352	388	339
305	Su	5 °C	1008	1010	1286	18	26	29	219	219	219	303	292	290
309	Su	5 °C	1026	1008	1298	20	28	30	243	247	230	338	352	304
311	Su	5 °C	1053	999	1201	20	28	30	254	260	249	348	359	331
315	Su	5 °C	960	1144	1163	23	28	30	252	266	251	362	338	330
320	Su	5 °C	937	1203	1279	24	28	30	270	236	221	379	311	284
303	Su	-8 °C (min)	998	1098	1269	18	27	29	172	226	212	202	290	271
308	Su	-8 °C (min)	1065	1033	1321	19	27	29	165	209	199	185	283	261
310	Su	-8 °C (min)	1114	947	1229	21	28	30	222	237	229	288	308	284
312	Su	-8 °C (min)	1092	1065	1261	23	28	30	209	257	233	255	320	301
313	Su	-8 °C (min)	913	1197	1279	25	28	31	227	194	214	271	245	273
317	Su	-8 °C (min)	931	1236	1263	25	28	31	219	196	228	283	253	290
205	Au	5 °C	1059	1059	1294	17	26	29	236	246	229	357	386	325
206	Au	5 °C	1029	1032	1312	18	26	29	259	255	248	406	400	350
211	Au	5 °C	1019	1059	1247	20	28	30	239	251	233	367	384	320
214	Au	5 °C	1078	932	1268	20	29	30	253	280	260	381	459	380
215	Au	5 °C	995	1137	1278	23	28	30	247	259	236	375	381	328
217	Au	5 °C	919	1129	1228	24	28	30	263	270	259	442	403	370
203	Au	-8 °C (min)	1027	1040	1233	18	27	29	170	227	218	205	313	299
207	Au	-8 °C (min)	1017	1043	1310	19	27	30	227	238	221	351	374	314
208	Au	-8 °C (min)	1055	922	1261	21	28	30	230	258	228	348	399	315
212	Au	-8 °C (min)	1050	943	1218	22	28	30	254	248	237	403	388	337
213	Au	-8 °C (min)	898	1176	1272	25	28	31	234	221	211	360	307	283
218	Au	-8 °C (min)	942	1179	1302	25	28	31	245	224	243	349	318	324
402	SA	5 °C	1029	1132	1278	18	26	29	194	236	218	259	337	298
405	SA	5 °C	1014	1085	1332	18	26	29	271	292	261	385	426	356
406	SA	5 °C	1131	1017	1328	20	28	30	245	248	244	362	378	342
410	SA	5 °C	1096	1021	1236	21	28	30	264	306	263	403	464	355
414	SA	5 °C	934	1136	1209	23	28	31	269	240	255	378	331	342
420	SA	5 °C	974	1179	1209	24	28	30	262	254	258	398	352	336
401	SA	-8 °C (min)	1029	1128	1312	19	27	29	191	227	226	247	313	298
407	SA	-8 °C (min)	1007	1013	1236	19	28	30	191	210	231	253	282	312
409	SA	-8 °C (min)	1056	1046	1233	21	28	31	168	230	228	197	295	307
411	SA	-8 °C (min)	1094	1063	1279	23	28	30	210	232	223	263	296	286
419	SA	-8 °C (min)	918	1230	1291	25	29	31	233	229	245	294	286	312
421	SA	-8 °C (min)	1065	1303	1348	25	29	31	258	235	288	361	312	364

Measurement of F_o and F_m in March

at nearly unstressed state

No.	Treatment	t (°C)	$F_{o\,20}$	$F_{m\,20}$
502	C2	19	181	905
503	C2	22	166	743
505	C2	18	191	987
506	C2	21	172	829
509	C2	17	170	721
510	C2	22	173	853
511	C2	19	193	796
512	C2	21	171	718
513	C2	20	183	889
514	C2	23	197	929
521	C2	23	202	966
522	C2	18	170	701
601	Wi	22	185	908
602	Wi	21	183	721
604	Wi	23	162	717
605	Wi	23	163	688
608	Wi	17	205	879
610	Wi	20	201	947
613	Wi	21	199	1042
614	Wi	19	205	1021
615	Wi	22	187	886
618	Wi	20	183	907
619	Wi	18	201	962
621	Wi	19	205	1053
701	SW	22	188	881
702	SW	23	211	1107
703	SW	21	221	1157
704	SW	19	197	900
705	SW	23	234	1245
706	SW	23	187	839
708	SW	17	215	1101
710	SW	19	218	1149
715	SW	20	201	982
716	SW	21	230	1143
717	SW	21	207	1011
719	SW	18	230	1106
